# 

**Published:** 1862-10

**Authors:** 


### BOOKS RECIEVED.

*University of the Pacific; fifth Annual Announcement of the Medical Department, Session of* 1862–63; *also Valedictory Address delivered at the Public Commencement, held March* 15*th,* 1862, *by* HENRY GIBBONS, M. D., *Professor of Materia Medica. San Francisco,* 1862.

*Dislocation of the Femur into the Ischiatic Notch; reduction by Manipulation; Death from Rupture of the Bladder; Dissection of the Hip. By* JOSEPH C. HUTCHISON, M. D., *Professor of Operative Surgery and Surgical Anatomy, Long Island College Hospital; Surgeon to the Brooklyn City Hospital, etc. Albany:* CHARLES VAN BENTHUYSEN, 1862.

*An Address on the Life and Character of the late* CHARLES EDWARD ISAACS, M. D., *delivered to the Graduates of Long Island College Hospital, Brooklyn, N. Y., at the Annual Commencement, July* 14, 1862. *By* JOSEPH C. HUTCHISON, M. D., *Professor of Operative Surgery and Anatomy. New York,* 1862.

*Report of the Surgeons of the New York Ophthalmic Hospital, for the years* 1860–61, *with the Anniversary Address, by* J. L. KIERNAN, A. M., M. D., *with a Catalogue of the Students of the New York Ophthalmic School, from* 1852 *to* 1862.

*Variola; its Nature and Treatment, with an Addendum. By* ANDREW NEBINGER, M. D. *Philadelphia,* 1862.

